# Psychotic symptoms in eating disorders: a case report

**DOI:** 10.1192/j.eurpsy.2023.1798

**Published:** 2023-07-19

**Authors:** E. Arroyo Sánchez, P. Setién Preciados, A. Sanz- Giancola

**Affiliations:** 1 Psiquiatría, Hospital Universitario Príncipe de Asturias, Madrid, Spain

## Abstract

**Introduction:**

Psychotic symptoms and eating disorders can occur in the same person, sometimes at the same time. This comorbidity is not well studied despite the difficulties of management at both clinical and pharmacological levels that it may entail. We present the case of a 35-year.old female patient with anorexia nervosa with years of evolution, currently admitted to a center specializing in Eating Disorders, who comes to the emergency department with psychotic symptoms.

**Objectives:**

To know the prevalence of comorbidity of psychotic symptoms in people with eating disorders, as well as possible risk factors, severity and management of them.

**Methods:**

Presentation of a case and review of the available literature on the presence of symptoms of the psychotic sphere in persons diagnosed with eating disorders.

**Results:**

The literature reflects data of a prevalence of 10-15% of patients with eating disorders presenting psychotic symptoms. The presence of psychotic symptoms is not directly related to a greater severity of the eating disorder. Some genetic associations have been found, as well as alterations at the physiological, cognitive and brain structure level that coincide in both pathologies. In some cases, an improvement in eating behavior has been observed when the psychotic symptomatology is resolved. In the case of patients with bulimia nervosa, a higher number of psychotic symptomatology has been observed, such as paranoid ideations, which some studies relate to a greater emotional capacity and histrionic expressiveness of this patient profile.

**Conclusions:**

The comorbidity of psychotic symptoms and eating disorders is relatively frequent and makes us face challenges in the diagnosis, as well as in the management of these patients. This comorbidity is especially important in patients with bulimia nervosa. Future research is necessary to know a more exact management of these pathologies.

**Disclosure of Interest:**

None Declared

